# Isolation and Structure Elucidation of GM4-Type Gangliosides from the Okinawan Starfish *Protoreaster nodosus*

**DOI:** 10.3390/md10112467

**Published:** 2012-11-05

**Authors:** Ke Pan, Chiaki Tanaka, Masanori Inagaki, Ryuichi Higuchi, Tomofumi Miyamoto

**Affiliations:** Graduate School of Pharmaceutical Sciences, Kyushu University, Maidashi 3-1-1, Higashi-ku, Fukuoka 812-8582, Japan; Email: pan.ke.nj@gmail.com (K.P.); ctanaka@phar.kyushu-u.ac.jp (C.T.); inagaki@yasuda-u.ac.jp (M.I.); r-higuchi@daiichi-cps.ac.jp (R.H.)

**Keywords:** glycosphingolipid, ganglioside, GM4, starfish, *Protoreaster nodosus*

## Abstract

Three new ganglioside molecular species, termed PNG-1, PNG-2A, and PNG-2B were isolated from pyloric caeca of the starfish *Protoreaster nodosus*. Their structures were elucidated using a combination of spectroscopic and chemical methods, and characterized as 1-*O*-[8-O-methyl-N-acetyl-α-neuraminosyl-(2→3)-β-galactopyranosyl]-ceramide for PNG-1, 1-*O*-[β-galactofuranosyl-(1→3)-α-galactopyranosyl-(1→4)-8-*O*-methyl-*N*-acetyl-α-neuraminosyl-(2→3)-β-galactopyranosyl]-ceramide for PNG-2A, and 1-*O*-[β-galactofuranosyl-(1→3)-α-galactopyranosyl-(1→9)-*N*-acetyl-α-neuraminosyl-(2→3)-β-galactopyranosyl]-ceramide for PNG-2B. PNG-2A and PNG-2B represent the first GM4 elongation products in nature.

## 1. Introduction

Gangliosides, sialic acid-containing glycosphingolipids (GLSs), are expressed in the outer leaflet of the plasma membrane of all vertebrate cells. Since the structure of the first mammalian ganglioside GM1 was described in 1963, a great deal of studies have been devoted to fully understand the ganglioside structural complexity, metabolism, cellular topology, biological functions, and pathobiological implications [1,2]. Ganglioside research is still far from being considered concluded, but there is a general agreement to consider gangliosides as functional molecules involved in the modulation of enzyme properties, cell signaling, cell adhesion and protein sorting [3,4]. 

In contrast to their ubiquity in vertebrates, gangliosides are currently believed to be only present in the phylum Echinodermata within invertebrates. During the past two decades, over 40 gangliosides were obtained from diverse echinoderm species in our laboratory, and some of them exhibited potent neuritogenic activities toward the neuron-like rat adrenal pheochromocytoma cell lines (PC-12) through nerve cell growth factors (NGFs) [5,6]. With regard to starfish gangliosides, the basic structure of the sugar part is lactose, and the sugar moieties are classiﬁed into two types, either sialic acids [*N*-acetylneuraminic acid (NeuAc), *N*-glycolylneuraminic acid (NeuGc)] or *N*-acetylgalactosamine (GalNAc) attached to C-3 of galactose (Gal) in the lactose moiety [6]. 

Recently, we reported the identification of a galactocerebroside molecular species (PNC-1) from the starfish *P. nodosus* [7]. In a continuing investigation into GLS constituents of this animal, we isolated three new ganglioside molecular species and describe their structures in the present article. 

## 2. Results and Discussion

**Figure 1:**
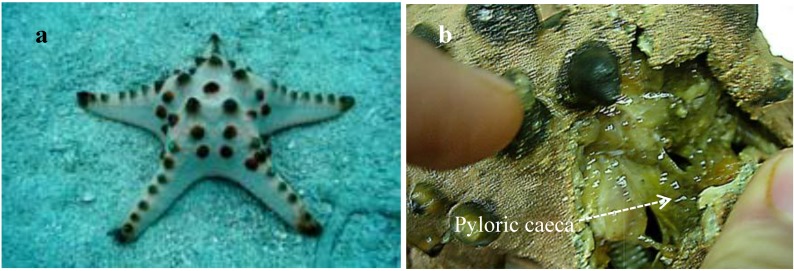
(**a**) *Protoreaster nodosus* and (**b**) Pyloric caeca of *P. nodosus.*

Pyloric caeca, carefully dissected from fresh materials (Figure 1), were extracted with CHCl_3_/MeOH. The combined extract was subjected to successive silica gel, RP-8, and Sephadex LH-20 column chromatography to give three ganglioside molecular species named PNG-1, PNG-2A, and PNG-2B, respectively. These three ganglioside molecular species showed a single spot and positive reaction with resorcinol reagent on normal-phase thin layer chromatography (TLC) plates.

### 2.1. Core Structure of PNG-1

**Figure 2:**
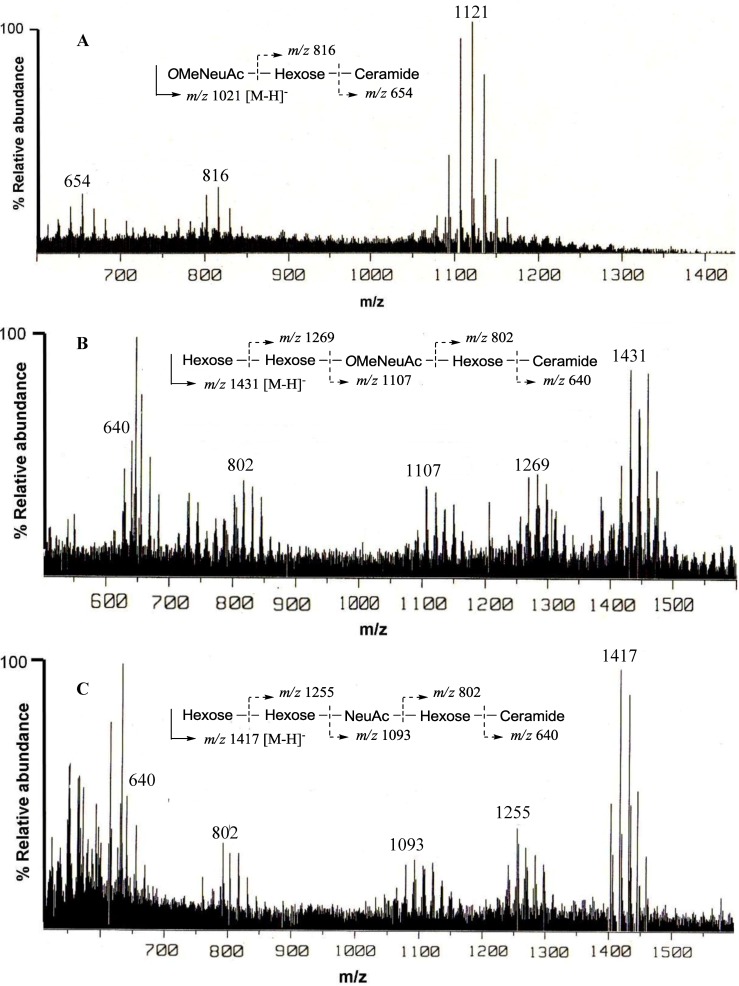
Negative ion FAB-MS spectra of PNG-1 (**A**), PNG-2A (**B**) and PNG-2B (**C**) and their major fragment ion peaks due to cleavage of the glycosidic linkages.

The negative FAB-MS spectrum of PNG-1 showed a series of pseudo-molecular ion peaks [M − H]^−^ at *m/z* 1079–1149. The fragment ion peaks arising from cleavage of the glycosidic linkages of the major component (*m/z* 1121) were observed at *m/z* 816 [M − H − 305; A] and 654 [A − 162; ceramide] as shown in Figure 2A. These data indicated that PNG-1 contained a disaccharide (OMeNeuAc-Hexose-Ceramide). The ^1^H-NMR spectrum of PNG-1 exhibited a strong broad signal due to the aliphatic methylenes at *δ*_H_ 1.22, a terminal methyl signal at *δ*_H_ 0.80 and several multiplets between *δ*_H_ 4.00 and 4.80 due to oxygenated methine and methylene protons. These results indicated that PNG-1 was a glycosphingolipid. Some typical signals of a Neu5Ac, such as *δ*_H_ 2.28 (t, *J *= 11.4 Hz, H*_ax_* − 3) and 3.43 (br.d, *J *= 8 Hz, H*_eq_*− 3), were also detected, suggesting PNG-1 was a ganglioside. 

Furthermore, the Neu5Ac was probably methylated, since a methoxy singlet at *δ*_H_ 3.83 (3H, s) was observed. In the ^13^C-NMR spectrum, two anomeric carbon signals were clearly observed, and one of them (appearing at *δ*_C_ 100.7) was thought to be the C-2 signal of Neu5Ac.

**Figure 3:**
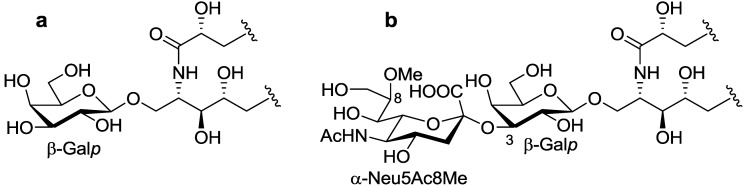
The core structures of Gangliosides (GLSs) from *P. nodosus*. (**a**) PNC-1 and (**b**) PNG-1.

The ceramide moiety of PNG-1 consisted of α-hydroxy fatty acids (FAs) and phytosphingosine-type long chain bases (LCBs). In the COSY spectrum, taking H-2 of the LCB as the starting point, a structural fragment from C-1 to C-6 was established. Another fragment in FA, from C-2 to C-4, was also connected by extensive analysis of COSY and TOCSY spectra. The stereochemistry of the ceramide moiety was presumed to be identical to that of PNC-1 (Figure 3a), which was isolated from the same material [7].

The structure of the sugar moiety was determined as follows. The ^1^H- and ^13^C-NMR data (Table 1) showed that PNG-1 contained a disaccharide moiety consisting of galactopyranose (Gal*p*) and mono-methylated Neu5Ac. Identification of the neutral sugar as Gal was also supported by sugar composition analysis using GC. The β-configuration of the Gal*p* unit was determined by the *J*-value of the anomeric proton [*δ*_H_ 4.79 (d, *J* = 7.9 Hz)]. The configuration of the Neu5Ac moiety was presumed to be α-configuration, based on the chemical shifts of the anomeric carbon at *δ*_C_ 100.7 and H-3 methylene protons [8]. 

**Table 1:** ^1^H- and ^13^C-NMR data of PNG-1, PNG-2A and PNG-2B.

Position			PNG-1		PNG-2A		PNG-2B	
			*δ*_H_^a^	*δ*_C_	*δ*_H_^a^	*δ*_C_^c^	*δ*_H_^a^	*δ*_C_^c^
LCB		1a	4.60 (m)	68.9 (t)	4.60 ^b^	68.8 (t)	4.21^ b^	69.0 (t)
		1b	4.18 ^b^		4.20 (m)		4.62 ^b^	
		2	4.81 (br.s)	50.6 (d)	4.82 (br.s)	50.8 (d)	4.83 (br.s)	51.0 (d)
		3	4.31 (br.s)	73.8 (d)	4.28 ^b^	74.0 (d)	4.30 ^b^	73.8 (d)
		4	4.08 (m)	71.6 (d)	4.08 ^b^	71.9 (d)	4.09 ^b^	71.7 (d)
		5a	1.96 (m)	31.5 (t)	1.97 (2H) ^b^	32.3 (t)	1.98 ^b^	32.0 (t)
		5b	1.85 (m)				1.86 ^b^	
		6a	1.74 (m)	26.1 (t)	1.73 (m)	25.5 (t)	1.60(2H, m)	25.0 (t)
		6b	1.52 (m)		1.50 (m)			
FA		1		175.7 (s)		n.d.		173.9 (s)
		2	4.49 ^b^	71.7 (d)	4.50 ^b^	71.6 (d)	4.51 ^b^	71.9 (d)
		3a	1.98 (m)	34.9 (t)	1.98 ^b^	35.2 (t)	2.02 (m)	34.7 (t)
		3b	1.90 (m)		1.90 ^b^		1.90 (m)	
		4	1.56 (2H, m)	25.1 (t)	1.58 (2H, m) ^b^	25.0 (t)	1.75 (m)	26.2 (t)
							1.52 (m)	
β-Gal*p*	1		4.79 (d, 7.9)	104.0 (s)	4.79 (d, 7.9)	104.5 (d)	4.74^ b^	104.0 (d)
	2		4.24 (t, 8.3)	69.8 (d)	4.22 ^b^	70.8 (d)	4.22 ^b^	69.7 (d)
	3		4.70 ^b^	76.7 (d)	4.70 ^b^	77.2 (d)	4.70 ^b^	76.8 (d)
	4		4.70 (br.s)	67.7 (d)	4.70 ^b^	68.6 (d)	4.64 ^b^	67.5 (d)
	5		4.04 (m)	75.6 (d)	4.04 ^b^	76.2 (d)	3.93 (br.s)	75.5 (d)
	6a		4.13 ^b^	61.0 (t)	4.39 ^b^	61.9 (t)	4.19 ^b^	61.3 (t)
	6b		4.38 (d, 11.4)		4.14 ^b^		4.10 ^b^	
α-Neu5Ac	1			173.9 (s)		n.d.		173.7 (s)
	2			100.7 (s)		n.d.		n.d.
	3eq		3.43 (br.d, 8.0)	41.6 (t)	3.43 (d)	n.d.	3.54 (br.d, 7.9)	41.5 (t)
	3ax		2.28 (t, 11.4)		2.15		2.28 (t, 11.0)	
	4		4.46 ^b^	68.3 (d)	4.50 ^b^	72.8 (d)	4.45 ^b^	68.2 (d)
	5		4.48 ^b^	53.2 (d)	4.70 ^b^	50.3 (d)	4.32 ^b^	53.2 (d)
	6		4.63 ^b^	73.8 (d)	4.69 ^b^	73.3 (d)	4.30 ^b^	71.5 (d)
	7		4.15 ^b^	68.4 (d)	4.22 ^b^	68.5 (d)	4.27 ^b^	68.5 (d)
	8		4.07 ^b^	81.4 (d)	4.06 ^b^	82.0 (d)	4.70^ b^	70.6 (d)
	9		4.15 (2H) ^b^	61.4 (t)	4.38 ^b^	61.9 (t)	4.38 (d, 7.2)	68.2 (t)
					4.10 ^b^		3.88 (d, 8.4)	
	10			173.7 (s)		n.d.		173.7 (s)
-NHAc	11		2.02 (s)	22.3 (q)	2.20 (s)	23.0 (q)	2.25 (s)	22.6 (q)
-OMe	12		3.83 (s)	58.1 (q)	3.79 (s)	58.2 (q)		
α-Gal*p*	1				5.50 (s)	96.6 (d)	5.22 (s)	98.9 (d)
	2				4.43 ^b^	67.8 (d)	4.53 ^b^	68.3 (d)
	3				4.36 ^b^	77.0 (d)	4.53 ^b^	78.0 (d)
	4				4.58 ^b^	69.7 (d)	4.68 ^b^	69.5 (d)
	5				4.36 ^b^	71.5 (d)	4.32 ^b^	71.5 (d)
	6				4.22 ^b^	61.9 (t)	4.14 (2H, m)	61.3 (t)
					4.06 ^b^			
β-Gal*f*	1				5.86 (s)	110.5 (d)	5.82 (s)	110.0 (d)
	2				4.74 ^b^	82.5 (d)	4.75 ^b^	81.8 (d)
	3				4.68 ^b^	78.3 (d)	4.67 ^b^	77.8 (d)
	4				4.71 ^b^	84.8 (d)	4.72 ^b^	84.5 (d)
	5				4.29 ^b^	71.8 (d)	4.26 ^b^	71.6 (d)
	6				4.06 (2H) ^b^	63.2 (t)	4.06 (2H) ^b^	63.5 (t)

^a^ Multiplicities and coupling constants are in parentheses; ^b^Obscured by other signals; ^c^ Assignments were based on HSQC experiment.

**Figure 4:**
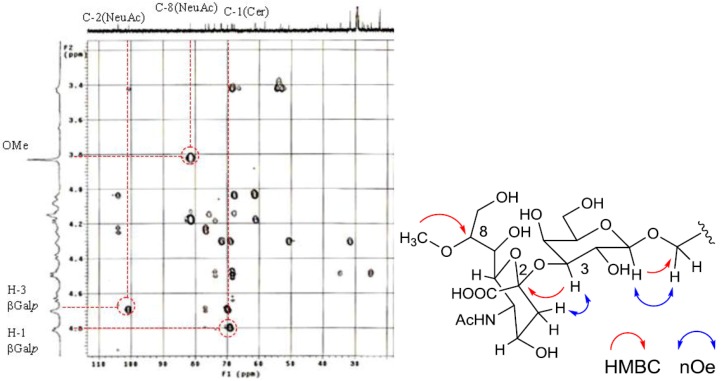
Partial HMBC spectrum and key HMBC and nOe correlations of PNG-1.

The position of the methoxyl group was determined to be at C-8 of Neu5Ac by HMBC correlation from the methyoxy proton (*δ*_H_ 3.83) to C-8 (*δ*_C_ 81.4). The linkage of the sugar moiety was established by HMBC and nOe correlations (Figure 4). Neu5Ac8Me was linked to C-3 of Gal*p* by the HMBC correlation from H-3 (*δ*_H_ 4.70) of Gal to C-2 (*δ*_C_ 100.7) of Neu5Ac8Me, as well as nOe correlation between H-3 (*δ*_H_ 2.28) of Neu5Ac8Me and H-3 of Gal*p*. In addition, Gal*p* was attached to C-1 of LCB by the HMBC correlations of H-1 (*δ*_H_ 4.79) of Gal*p* and C-1 (*δ*_C_ 68.9) of LCB, and of H-1 (*δ*_H_ 4.60) of LCB and C-1 (*δ*_C_ 104.0) of Gal*p*. Accordingly, PNG-1 was characterized to be 1-*O*-[8-O-methyl-N-acetyl-α-neuraminosyl-(2→3)-β-galactopyranosyl]-ceramide (Figure 3b).

### 2.2. Core Structure of PNG-2A

^1^H- and ^13^C-NMR spectra of PNG-2A showed signals characteristic of gangliosides. The negative FAB-MS spectrum of PNG-2A showed a series of pseudo-molecular ion peaks [M − H]^−^ at *m/z* 1400–1500. The fragment ion peaks arising from cleavage of the glycosidic linkages of the major component (*m/z* 1431) were observed at *m/z* 1269 [(M − H)^−^ − 162; A], 1107 [A − 162; B], 802 [B − 305; C], and 640 [C − 162; ceramide] as shown in Figure 2B. These data indicated the sequence of the oligosaccharide of PNG-2A could be depicted as Hexose-Hexose-*O*MeNeuAc-Hexose-Ceramide. In the ^1^H-NMR spectrum, three anomeric proton signals were clearly observed at *δ*_H_ 4.79 (d, *J* = 7.9 Hz), 5.50 (s), and 5.86 (s). Further, a singlet at *δ*_H_ 3.79 (3H, s) implied that the NeuAc moiety in PNG-2A was probably methylated.

**Figure 5:**
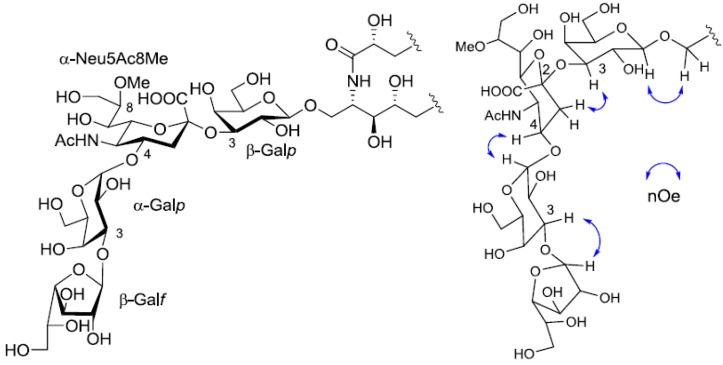
The core structure of PNG-2A and key nOe correlations.

Extensive analysis of COSY, TOCSY, and HSQC spectra led to the establishment of two partial structures in the ceramide moiety (from C-1 of LCB to C-6 of LCB and from C-2 of FA to C-4 of FA), suggesting that PNG-2A was composed of α-hydroxy fatty acid and phytosphingosine-type LCB. The stereochemistry of the ceramide moiety of PNG-2A was presumed to be identical with that of PNC-1 isolated from the same material. The structure of the sugar moiety was characterized as follows. To determine the sugar composition, PNG-2A was subjected to methanolysis. Comparison of the GC retention times of TMS ethers of methyl glycosides obtained from PNG-2A with those from standard hexoses showed that all the neutral sugars in PNG-2A were Gals. ^1^H- and ^13^C-NMR data of the sugar moiety were assigned as shown in Table 1. ^1^H-^1^H COSY correlations from the three anomeric proton signals revealed a galactose each for β-galactopyranose(β-Gal*p*), α-galactopyranose(α-Gal*p*), and β-galactofuranose(β-Gal*f*). In the ^13^C-NMR spectrum of PNG-2A, C-1, C-2 and C-3 carbon signals were not confirmed. The other proton and carbon signals were indicative of α-Neu5Ac8Me, the same as found in PNG-1. The sugar linkage was examined by NOESY spectrum. The nOe correlations between H-1 of β-Gal*f* and H-3 of α-Gal*p*, H-1 of α-Gal*p* and H-3 of α-Neu5Ac8Me, H-3 of α-Neu5Ac8Me and H-3 of β-Gal*p* were clearly observed. Accordingly, the core structure of PNG-2A was determined to be 1-*O*-[β-galactofuranosyl-(1→3)-α-galactopyranosyl-(1→4)-8-*O*-methyl-*N*-acetyl-α-neuraminosyl-(2→3)-β-galactopyranosyl]-ceramide (Figure 5).

### 2.3. Core Structure of PNG-2B

The negative FAB-MS spectrum of PNG-2B showed a series of pseudo-molecular ion peaks [M − H]^−^ at *m/z* 1400–1500. The fragment ion peaks arising from cleavage of the glycosidic linkages of the major component (*m/z* 1417) were observed at *m/z* 1255, 1093, 802 and 640. These data indicated that PNG-2B contained the same oligosaccharide sequence as PNG-2A. However, the fragment loss (291 mass) from *m/z* 1083 to *m/z* 902 suggested the existence of non-substituted Neu5Ac. Hence, the oligosaccharide moiety of PNG-2B could be depicted as Hexose-Hexose-NeuAc-Hexose-Ceramide as shown in Figure 2C. In the ^1^H-NMR spectrum, two anomeric proton signals were clearly observed at *δ*_H_ 5.82 (s) and 5.22 (s), and another anomeric proton signal, obscured by other resonances, was readily discernable at *δ*_H_ 4.74 by HSQC spectrum analysis. Further, the absence of methoxy methyl signals confirmed that the NeuAc moiety in PNG-2B was not methylated.

**Figure 6:**
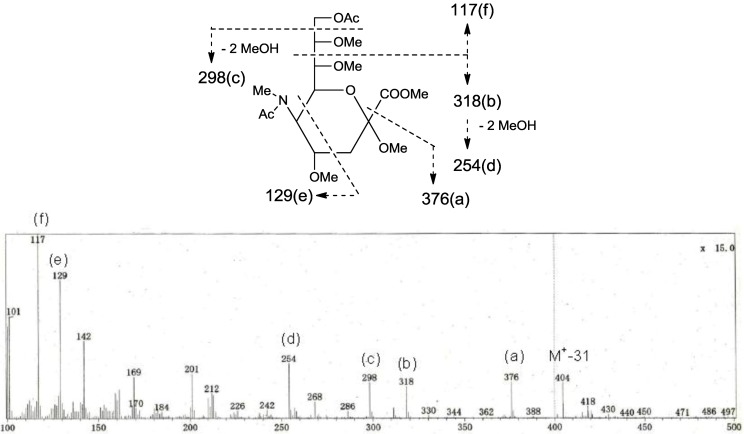
EI-MS spectrum of the acetate of partially methylated NeuAc derived from 9-linked Neu5Ac and the assignment of its valuable fragment ion peaks.

**Figure 7:**
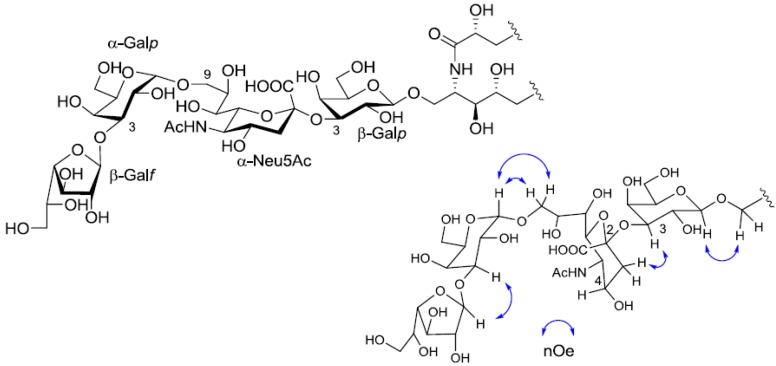
The core structure of PNG-2B and key nOe correlations.

The structure of the ceramide moiety was identified as α-hydroxy FA and phytosphingosine-type LCB by comparing the ^13^C-NMR, COSY and TOCSY spectra with those of PNG-1 and PNG-2A. The structure of the tetrasaccharide moiety was characterized as follows. ^1^H-^1^H COSY, TOCSY and HSQC spectra revealed the correlation of β-Gal*p*, α-Gal*p*, α-Neu5Ac and β-Gal*f* (Table 1). The linkage of each monosaccharide was examined by NOESY experiment. The nOe correlations between H-1 of β-Gal*f* and H-3 of α-Gal*p*, H-1 of α-Gal*p* and H-9 of α-Neu*p*5Ac, H-3 of α-Neu*p*5Ac and H-3 of β-Gal*p* suggested the sugar moiety was β-Gal*f*-(1→3)-α-Gal*p*-(1→9)-α-Neu5Ac-(2→3)-β-Gal*p*. The structure of the oligosaccharide moiety was also confirmed by chemical methods. PNG-2B was subjected to methanolysis. Comparison of the GC retention times of TMS ethers of methyl glycosides obtained from PNG-2B with those from standard Gals showed that all the neutral sugars in PNG-2B were Gals. To verify the sugar linkages, PNG-2B was subjected to methylation analysis. Partially methylated alditol acetates prepared from the permethylated PNG-2B were characterized as the alditols derived from terminal Gal*f* and 3-linked Gal*p* in a ratio of approx. 1:2 by GC-MS analysis. The acetate of partially methylated Neu5Ac derived from 9-linked Neu5Ac was detected (Figure 6). These observations unambiguously confirmed the proposed structure of the tetrasaccharide moiety. Therefore, the core structure of PNG-2B was established as 1-*O*-[β-galactofuranosyl-(1→3)-α-galactopyranosyl-(1→9)-*N*-acetyl-α-neuraminosyl-(2→3)-β-galactopyranosyl]-ceramide (Figure 7).

### 2.4. Ceramide Compositions of PNG-1, PNG-2A and PNG-2B

PNG-1, PNG-2A and PNG-2B were each subjected to methanolysis and periodate oxidation as previously described for PNC-1 [7]. FAs and LCBs were analyzed by GC-MS as fatty acid methyl esters (FAMEs) and long chain aldehydes (LCAs), respectively. The results were summarized in Table 2 and Table 3.

**Table 2:** Fatty acid composition of PNG-1, PNG-2A and PNG-2B.

Fatty acid methyl ester	Composition [%]		
	PNG-1	PNG-2A	PNG-2B
Methyl 2-hydroxyicosanoate (*normal*-C20)	3.3	4.0	5.0
Methyl 2-hydroxyhenicosanoate (*normal*-C21)	1.9	5.3	5.9
Methyl 2-hydroxydocosanoate (*normal*-C22)	40.4	39.0	39.5
Methyl 2-hydroxytricosanoate (*normal*-C23)	26.4	23.5	24.0
Methyl 2-hydroxytetracosanoate (*normal*-C24)	28.1	28.2	25.6

**Table 3:** Long chain base composition of PNG-1, PNG-2A and PNG-2B.

Long chain aldehyde	Parent LCB *	Composition [%]		
		PNG-1	PNG-2A	PNG-2B
tridecanal (*normal*-C13)	*normal-*t16:0	14.7	13.8	19.9
12-methyltridecanal (*iso-*C14)	*iso-*t17:0	26.8	25.5	28.3
Tetradecanal (*normal*-C14)	*normal-*t17:0	9.4	8.5	10.6
13-methyltetradecanal (*iso*-C15)	*iso-*t18:0	28.9	28.3	26.1
12-methyltetradecanal (*anteiso*-C15)	*anteiso-*t18:0	4.2	4.7	4.0
pentadecanal (*normal*-C15)	*normal-*t18:0	7.0	12.7	5.4
13-methylpentadecanal (*anteiso*-C16)	*anteiso-*t19:0	9.0	6.5	5.7

* The chain length and number of double bonds are denoted with the prefix “t” to designate trihydroxy bases (phytosphingosine).

## 3. Experimental Section

### 3.1. Animal Material

*Protoreaster nodosus* was collected at Katsuren, the east coast of Okinawa, in June 2009. A voucher specimen was deposited in the Herbal Museum, Graduate School of Pharmaceutical Sciences, Kyushu University.

### 3.2. Isolation of Gangliosides

Pyloric caeca (600 g) dissected from 20 specimens of the starfish *Protoreaster nodosus* were extracted with CHCl_3_/MeOH (1:1, v/v, 2 L) for three times at room temperature. The extract was concentrated *in vacuo* to give a residue, which was subjected to silica gel column chromatography eluted with CHCl_3_/MeOH/H_2_O (10:1:0 to 6:4:1, v/v/v) to give four fractions. Fraction 3 and Fraction 4 were each further chromatographed on a LiChroprep RP-8 (Merck, Darmstadt, Germany) column (H_2_O/MeOH, 1:0 to 0:1, v/v). Fraction 3-4 containing crude, less polar ganglioside was finally purified by Sephadex LH-20 chromatography with CHCl_3_/MeOH (1:1, v/v) to afford PNG-1 (70.0 mg). Fraction 4-4 containing polar gangliosides was desalted on a Sephadex LH-20 column with CHCl_3_/MeOH (1:1, v/v) and subsequently subjected to repeated silica gel column chromatography to give PNG-2A (3.0 mg) and PNG-2B (7.0 mg). PNG-1, PNG-2A, and PNG-2B showed a positive reaction with resorcinol reagent on TLC plates.

### 3.3. FAB-MS

For FAB-MS analysis, a JEOL SX-102 mass spectrometer (JEOL, Tokyo, Japan) was used (Xenon atom beam, 5 kV; ion source accelerating potential 10 kV; matrix, hexamethylphosphoramide + glycerol for negative ion mode).

### 3.4. GC-MS

A ShimazuQP-5050A (SHIMADZU, Kyoto, Japan) instrument was used with the following settings: EI mode, ionizing potential of 70 eV; injection temperature, 300 °C; separation temperature, 280 °C; ion source temperature, 300 °C. A fused silica capillary column INERT CAP5 (0.25 mm I.D., 30 m, GLSciences INC., Tokyo, Japan) was used for gas-chromatographic separation with Helium as a carrier at a flow rate of 30 mL/min.

### 3.5. NMR Spectroscopy

Spectra were recorded on a Varian INOVA600 spectrometer. The operating conditions were as follows: ^1^H: frequency, 600 MHz; sweep width, 8 kHz; sampling point, 44 k; accumulation, 64 pulses; temperature, 30 or 40 °C. ^13^C: frequency, 150 MHz; sweep width, 32 kHz; sampling point, 160 k; accumulation, 10000 pulses; temperature, 30 °C. Chemical shifts were referenced to the residual solvent signals (*δ*_H_ 7.19, *δ*_C_ 123.5) in C_5_D_5_N and D_2_O (20/1, v/v). Conventional pulse sequences were used for DQF-COSY, NOESY, HSQC, and HMBC. The mixing time in the NOESY experiment was set to 500 ms. TOCSY spectra were acquired using the standard MLEV17 spin-locking sequence and 120 ms mixing time. All spectra were recorded using the phase-sensitive mode. The size of the acquisition data matrix was 204 × 256 words in f2 and f1, respectively. Zero filling up to 2 k in f1 was made prior to Fourier transformation. Sine-Bell or shifted sine-bell window functions, with the corresponding shift optimized for every spectrum, were used for resolution enhancement, and baseline correction was applied in both dimensions. Water suppression was carried out by selective pre-saturation placing the carrier on the solvent resonance.

### 3.6. Sugar Composition Analysis

Ganglioside (*ca*. 1.0 mg) was heated with 5% HCl/MeOH in a sealed tube at 80 °C for 18 h. The reaction mixture was extracted with *n*-hexane to extract FAMEs. The remaining MeOH layer was neutralized with Ag_2_CO_3_ and filtered. The dried MeOH layer was reacted with 1-(trimethylsilyl) imidazole/pyridine (1:1) for 20 min at 70 °C to give the TMS ether of methyl glycosides. The TMS ethers were then analyzed by GC-MS [column temperature 100–250 °C (rate of temperature increase: 5 °C/min)].

TMS ether of methyl glycoside from D-galactose: *t_R_* [min] = 6.7, 7.2 and 7.7, TMS ether of methyl glycoside from PNG-1: *t_R_* [min] = 6.7, 7.2 and 7.7, TMS ether of methyl glycoside from PNG-2A: *t_R_* [min] = 6.7, 7.2 and 7.7, TMS ether of methyl glycoside from PNG-2B: *t_R_* [min] = 6.7, 7.2 and 7.7.

### 3.7. Sugar Linkage Analysis

PNG-2B (*ca*. 0.5 mg) was added to a NaH-DMSO solution, and the mixture was stirred for 20 min. Then, CH_3_I was added to the above reaction mixture, which was stirred for another 20 min to yield permethylated ganglioside. Permethylated ganglioside was hydrolyzed with 90% HCOOH/10% TFA and subsequently reduced by NaBD_4_. The residue was heated with Ac_2_O/pyridine, and the mixture was extacted with CHCl_3_. The extract, containing partially methylated alditol acetates, was concentrated and subjected to GC-MS analysis using a constant column temperature of 175 °C. 1,3,5-tri-*O*-acetyl-2,4,6-tri-*O*-methylgalacitol from 3-linked Gal*p*, *t_R_* [min] = 15.5: 1,4-di-*O*-acetyl-2,3,5,6-tetra-*O*-methylgalacitol from terminal Gal*f*, *t_R_* [min] = 12.9.

Separately, permethylated ganglioside was methanolyzed with 10% HCl in MeOH. The reaction mixture was concentrated *in vacuo*, and the residue was heated with Ac_2_O/pyridine. The reaction mixture was subjected to GC-MS analysis of sialic acid using a constant column temperature of 175 °C. Methyl *N*-acetyl-9-*O*-acetyl-*N*-methyl-2,4,7,8-tetra-*O*-methylneuraminate from 9-linked NeuAc, *t_R_* [min] = 25.1.

### 3.8. Ceramide Composition Analysis

Ganglioside (*ca*. 0.1 mg) was heated with 5% HCl/MeOH (0.2 mL) in a sealed tube at 80 °C for 2 h. The reaction mixture was diluted with MeOH (0.3 mL) and extracted with *n*-hexane. The extract was concentrated *in vacuo* to give a mixture of FAMEs, which was subjected to GC-MS [column temperature 180–320 °C (rate of temperature increase: 4 °C/min)]. The remaining MeOH layer, which contained a mixture of lyso-cerebroside, free long chain base and methyl glycoside, was dried *in vacuo*. The resiude was dissolved in CH_2_Cl_2_ (0.1 mL), a silica gel-supported NaIO_4_ reagent (ca. 0.2 mg) was added, and the mixture was vigorously stirred at room temperature. After 30 min, the reaction mixture was filtered through a short silica gel column and washed with CH_2_Cl_2_. Removal of solvent from the filtrate afforded a mixture of long chain aldehydes (LCAs), which was subjected to GC-MS [column temperature 180–320 °C (rate of temperature increase: 4 °C/min)]. FAMEs; methyl 2-hydroxyicosanoate, *t_R_* [min] = 26.8: methyl 2-hydroxyhenicosanoate, *t_R_* [min] = 27.8: methyl 2-hydroxydocosanoate, *t_R_* [min] = 28.9: methyl 2-hydroxytricosanoate, *t_R_* [min] = 29.9: methyl 2-hydroxytetracosanoate, *t_R_* [min] = 30.8. LCAs; tridecanal, *t_R_* [min] = 14.4: 12-methyl tridecanal, *t_R_* [min] = 15.4: tetradecanal, *t_R_* [min] = 16.0: 13-methyl tetradecanal, *t_R_* [min] = 17.0: 12-methyltetradecanal, *t_R_* [min] = 17.1: pentadecanal, *t_R_* [min] = 17.5: 13-methyl pentadecanal, *t_R_* [min] = 18.6.

## 4. Conclusions

In conclusion, a GM4-type ganglioside (PNG-1), along with two GM4 elongation products (PNG-2A and PNG-2B), was identified from the starfish *P. nodosus*. In 1964, GM4 was first revealed as a minor ganglioside of human brain [9]. Later, its high content in human myelin was recognized [10]. Yu *et al.* subsequently identiﬁed GM4 as a speciﬁc marker for human myelin and oligodendroglial perikarya [11]. Besides being closely associated with myelin in neural tissues, GM4 has been shown to exhibit immunosuppressive activity by inhibiting T-cell proliferative responses to tetanus toxoid [12,13]. GM4 has also been shown to speciﬁcally interact with myelin basic protein and to protect gangliosides against neuraminidase [14]. 

GM4 has been reported to be present in other vertebrate sources, such as chicken thymus, chicken embryonic liver, rat kidney, shark liver, and red sea bream intestine [15,16,17,18,19]. A comparative study on GM4 distribution in livers of various vertebrates showed that GM4 was expressed mainly in lower animals, such as bony fish and frog liver [20]. To our knowledge, the existence of GM4 in invertebrates remains unknown. Therefore, the present study describing the first case of invertebrate GM4-type ganglioside has a wide significance from chemical, biological, and biosynthetic points of view.

The other two gangliosides, PNG-2A and PNG-2B, are also unusual. They are GM4 elongation products, which have not been identified in nature. Further, the tetrasaccharide moiety of PNG-2B possesses a rare glycosylation position of sialic acid, namely a hexose link to C-9 of the NeuAc residue. This linkage, to the best of our knowledge, has not been reported for a natural ganglioside so far. Since PNG-1 possesses a NeuAc8Me residue, it is of particular interest to observe that PNG-2B having NeuAc, but not PNG-2A bearing NeuAc8Me, is the major elongation product. The methylation of sialic acid at C-8 is mainly found in echinoderm species, but only in minute amounts in higher animals [21]. This is in contrast to *N*-acetyl hydroxylation and *O*-acetylation, which have been conserved from echinoderms to mammals [22]. Why this modification has largely been lost during evolution is unknown. Solving this question may be one of the challenges for future research. With regard to the function of this substitution, it can only be assumed that it influences the action of sialidases and the biosynthesis of specific glycan structures [23]. Schauer *et al.* postulated that 8-*O*-methylated sialic acids might represent a stop signal for oligosaccharide chain elongation in echinoderm glycoconjugates [24]. Our finding of PNG-2A and PNG-2B, therefore, provides evidence supporting such a speculation.

## Supplementary Files

Supplementary File 1: PDF-Document (PDF, 2480 KB)
