# Supplementary material for: Isolation and Structure Elucidation of GM4-Type Gangliosides from the Okinawan Starfish Protoreaster nodosus
Source: Mar Drugs. 2012 Nov 5;10(11):2467–80. doi: 10.3390/md10112467 (PMC3509529; doi:10.3390/md10112467)

Supplementary Information

Figure S1. ^{1}H-NMR of PNG-1 (600 MHz, C_5D_5N/D_2O, 20/1). P2
Figure S2. ^{13}C-NMR of PNG-1 (150 MHz, C_5D_5N/D_2O, 20/1). P3
Figure S3. ^{1}H-^{1}H COSY of PNG-1 (600 MHz, C_5D_5N/D_2O, 20/1). P4
Figure S4. NOESY of PNG-1 (600 MHz, C_5D_5N/D_2O, 20/1). P5
Figure S5. HSQC of PNG-1 (600 MHz, C_5D_5N/D_2O, 20/1). P6
Figure S6. HMBC of PNG-1 (600 MHz, C_5D_5N/D_2O, 20/1). P7
Figure S7. ^{1}H-NMR of PNG-2A (600 MHz, C_5D_5N/D_2O, 20/1). P8
Figure S8. ^{1}H-^{1}H COSY of PNG-2A (600 MHz, C_5D_5N/D_2O, 20/1). P9
Figure S9. NOESY of PNG-2A (600 MHz, C_5D_5N/D_2O, 20/1). P10
Figure S10. HSQC of PNG-2A (600 MHz, C_5D_5N/D_2O, 20/1). P11
Figure S11. ^{1}H-NMR of PNG-2B (600 MHz, C_5D_5N/D_2O, 20/1). P12
Figure S12. ^{13}C-NMR of PNG-2B (150 MHz, C_5D_5N/D_2O, 20/1). P13
Figure S13. ^{1}H-^{1}H COSY of PNG-2B (600 MHz, C_5D_5N/D_2O, 20/1). P14
Figure S14. NOESY of PNG-2B (600 MHz, C_5D_5N/D_2O, 20/1). P15
Figure S15. HSQC of PNG-2B (600 MHz, C_5D_5N/D_2O, 20/1). P16
Figure S16. HMBC of PNG-2B (600 MHz, C_5D_5N/D_2O, 20/1). P17

Figure S1. ^{1}H-NMR of PNG-1 (600 MHz, C_5D_5N/D_2O, 20/1).

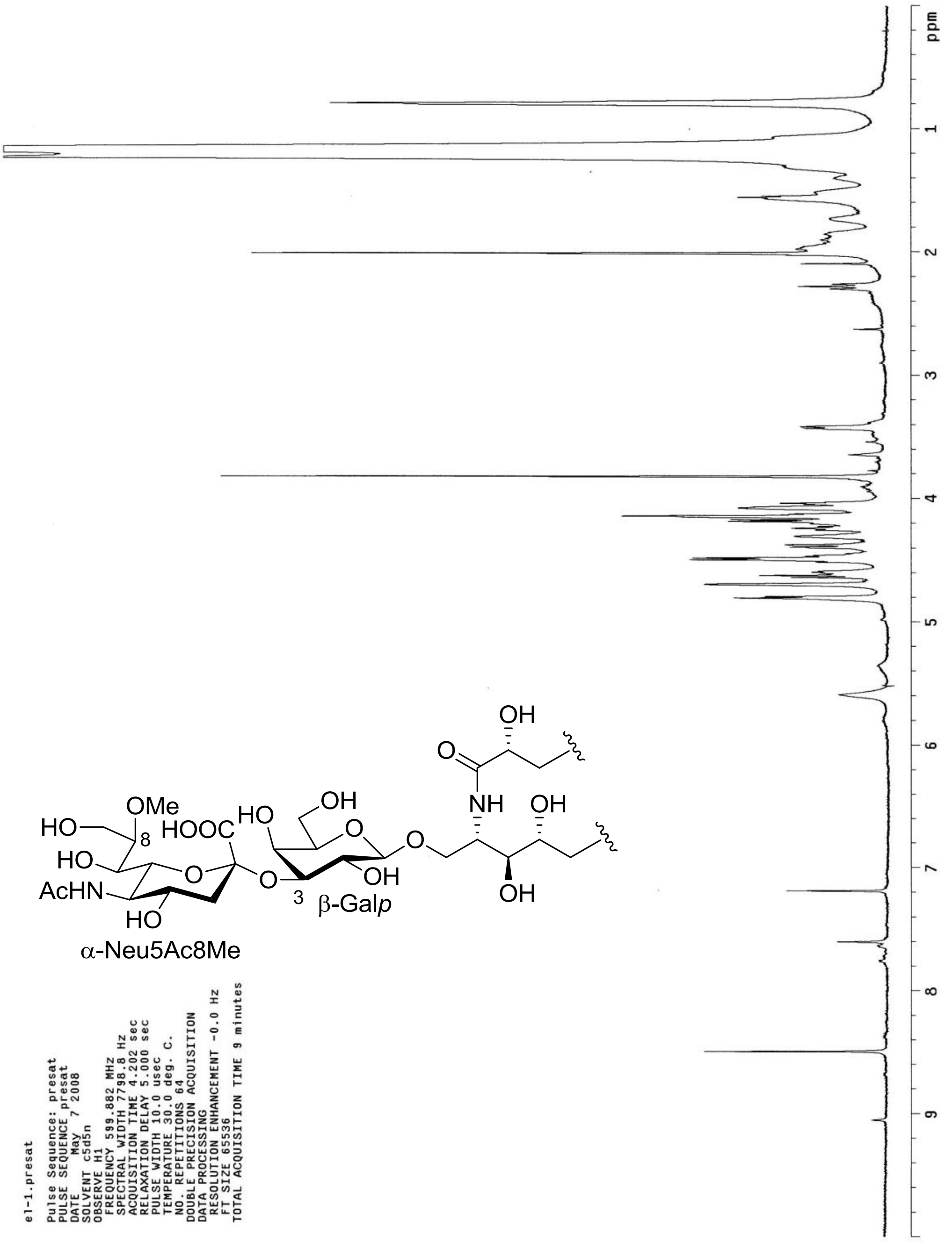

Figure S2. 13C-NMR of PNG-1 (150 MHz, C5D5N/D2O, 20/1).

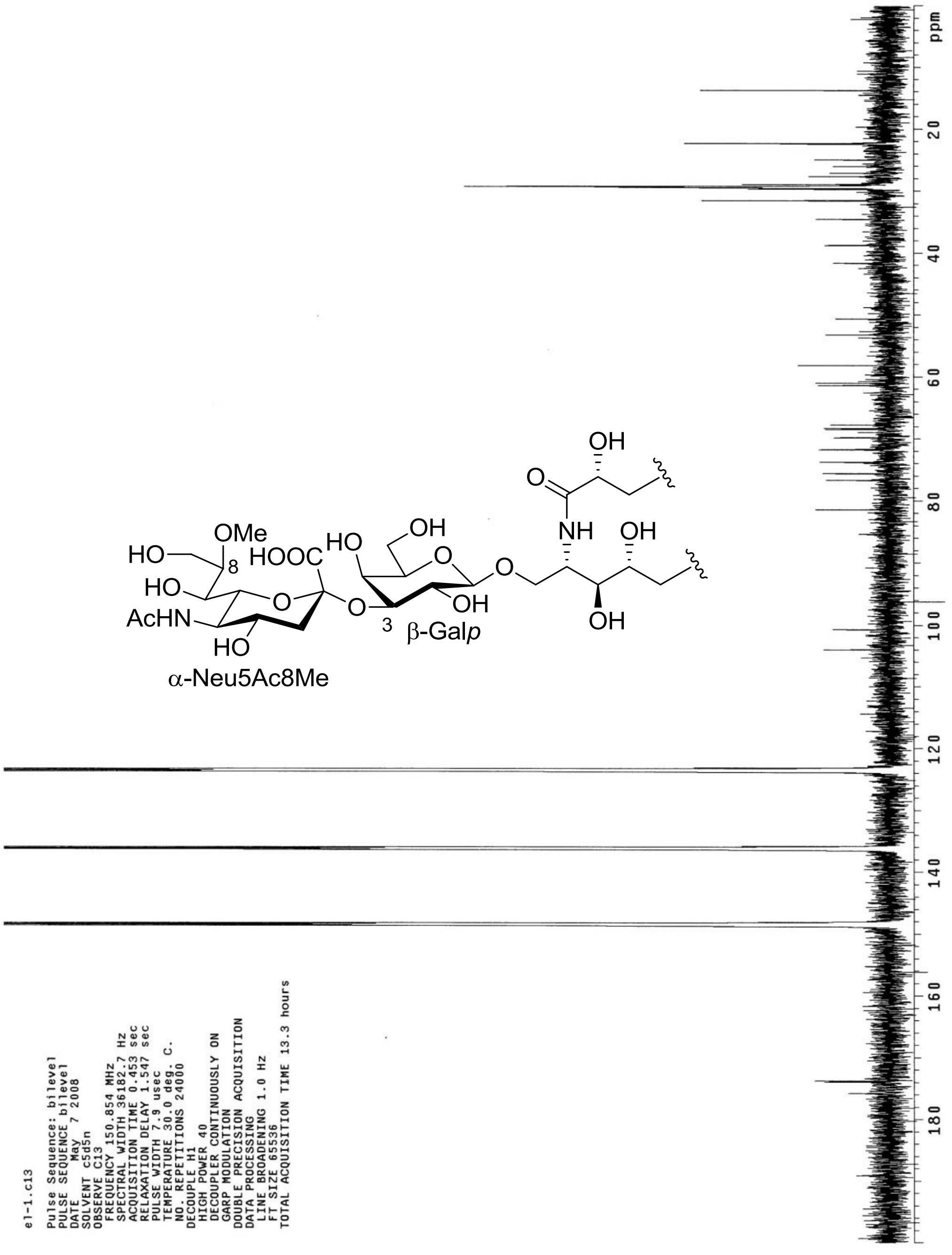

Figure S3. 1H-1H COSY of PNG-1 (600 MHz, C5D5N/D2O, 20/1).

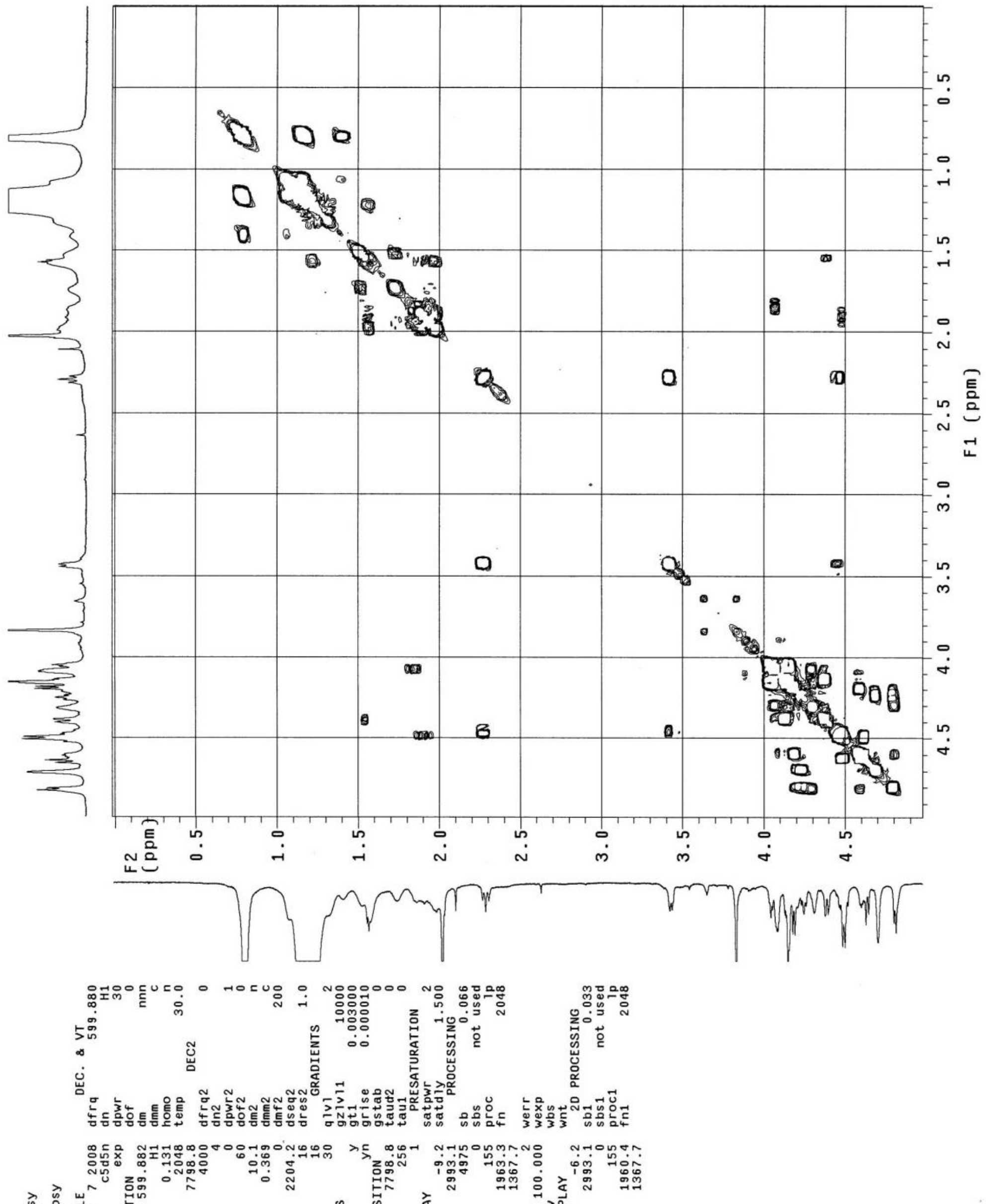

Figure S4. NOESY of PNG-1 (600 MHz, C_5D_5N/D_2O, 20/1).

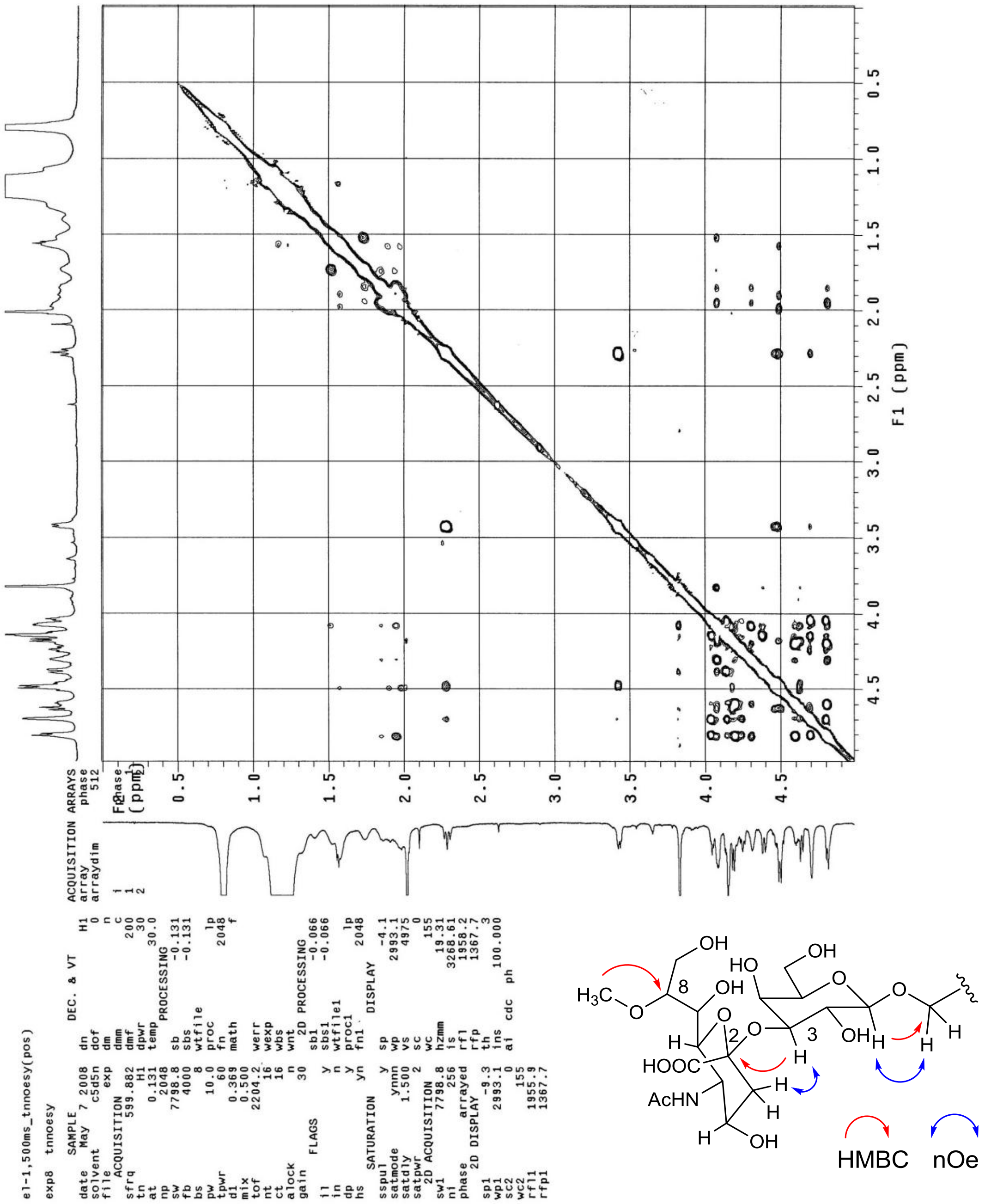

Figure S5. HSQC of PNG-1 (600 MHz, C_5D_5N/D_2O, 20/1).

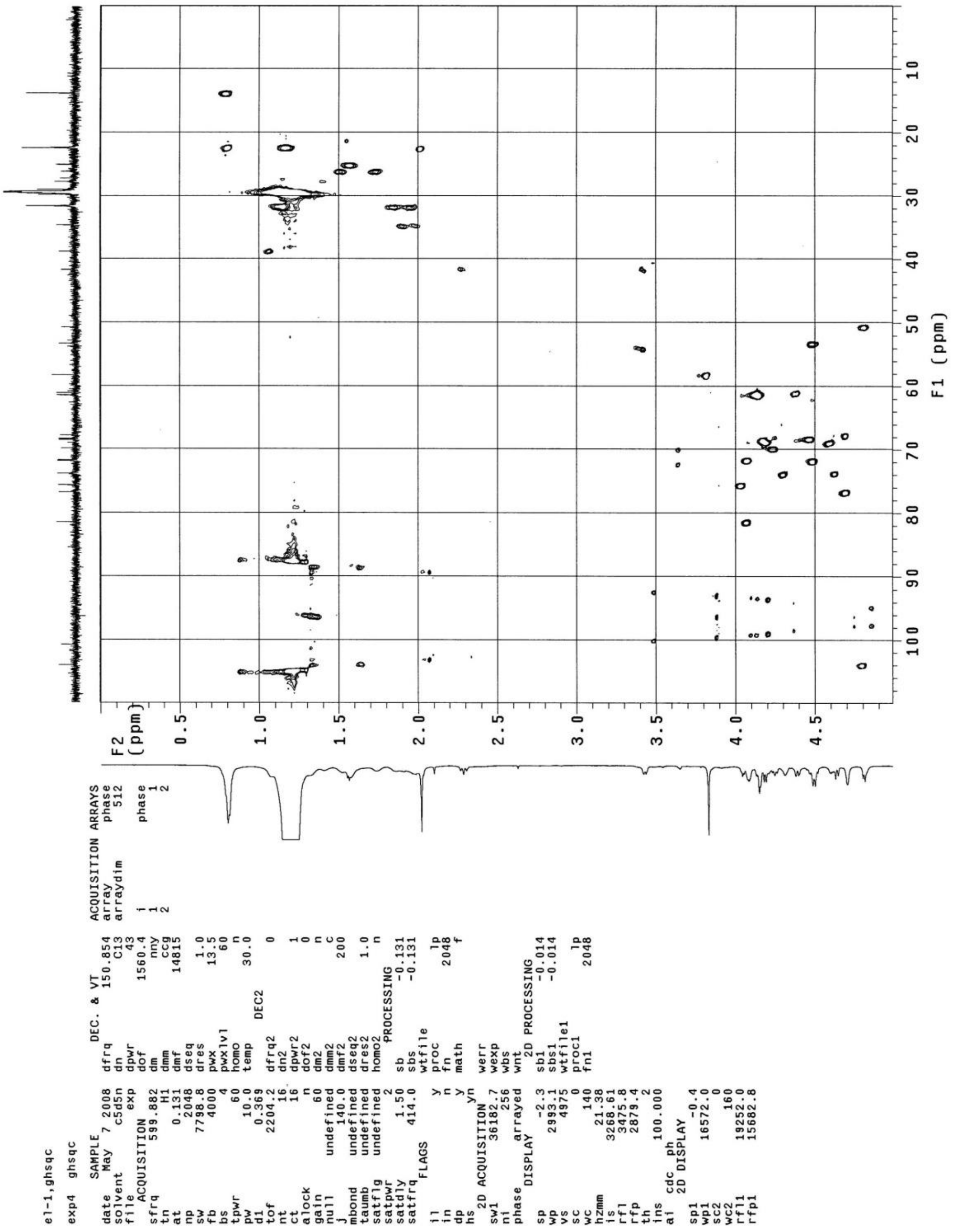

Figure S6. HMBC of PNG-1 (600 MHz, C_5D_5N/D_2O, 20/1).

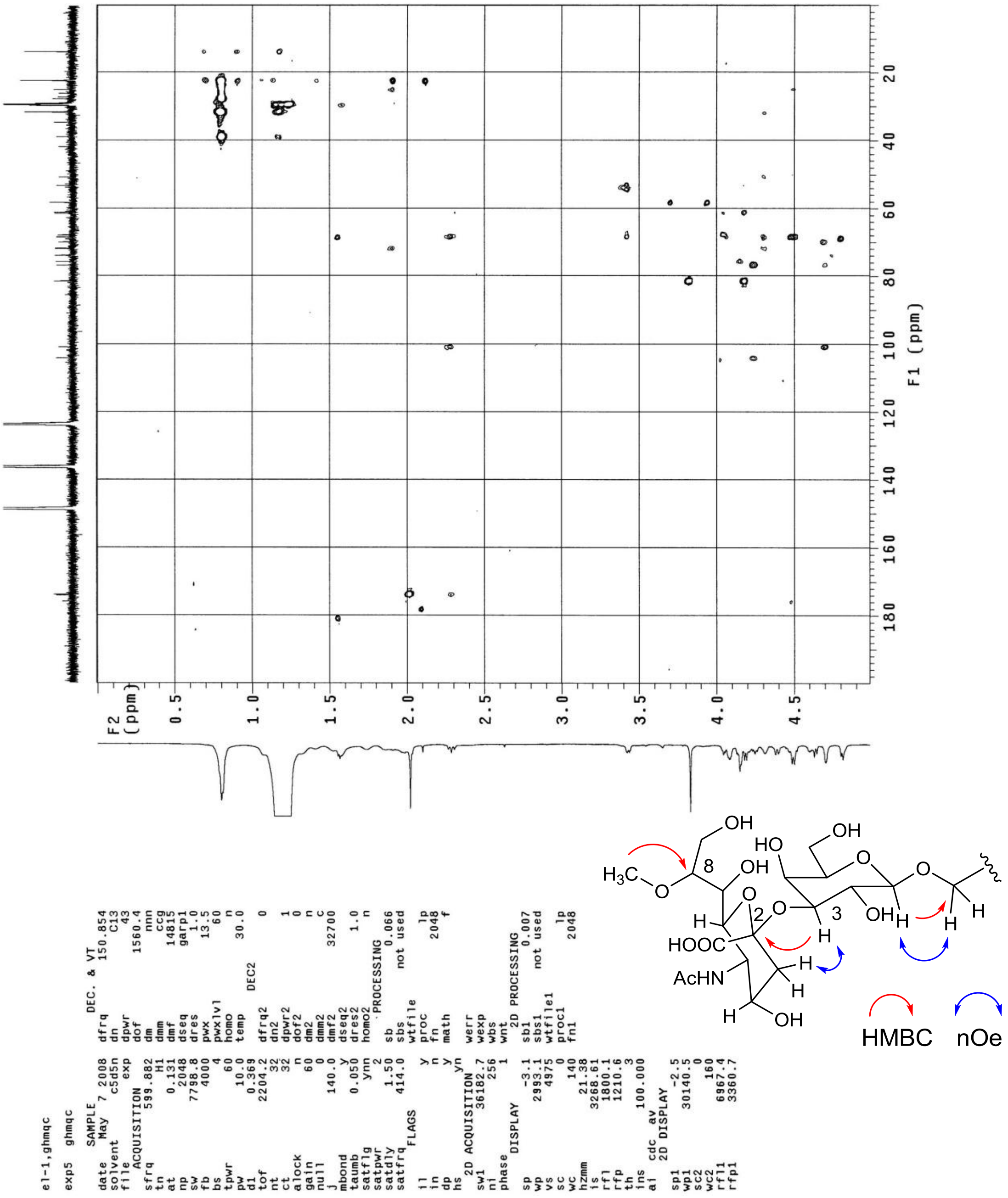

Figure S7. ^{1}H-NMR of PNG-2A (600 MHz, C_5D_5N/D_2O, 20/1).

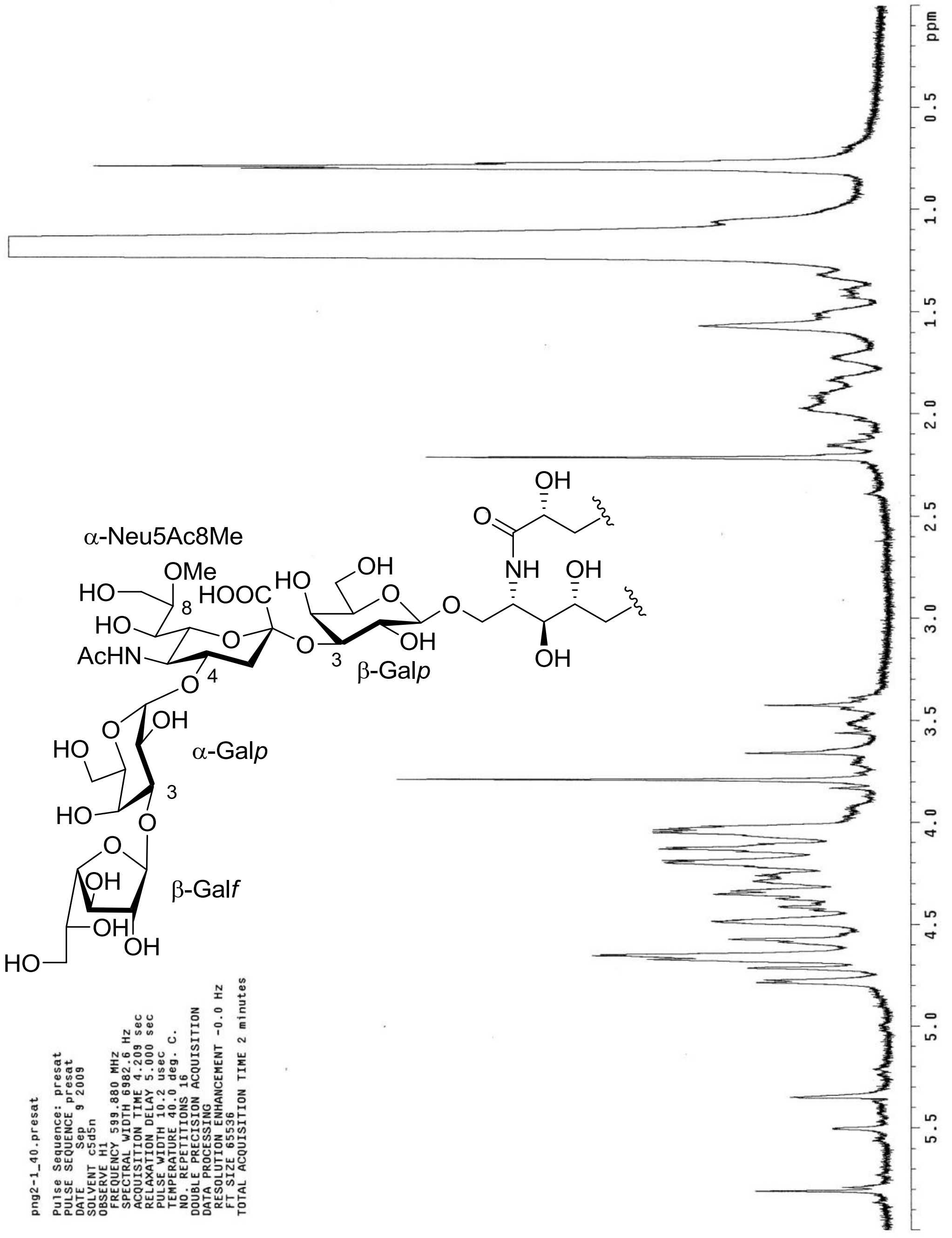

Figure S8. COSY of PNG-2A (600 MHz, C_5D_5N/D_2O, 20/1).

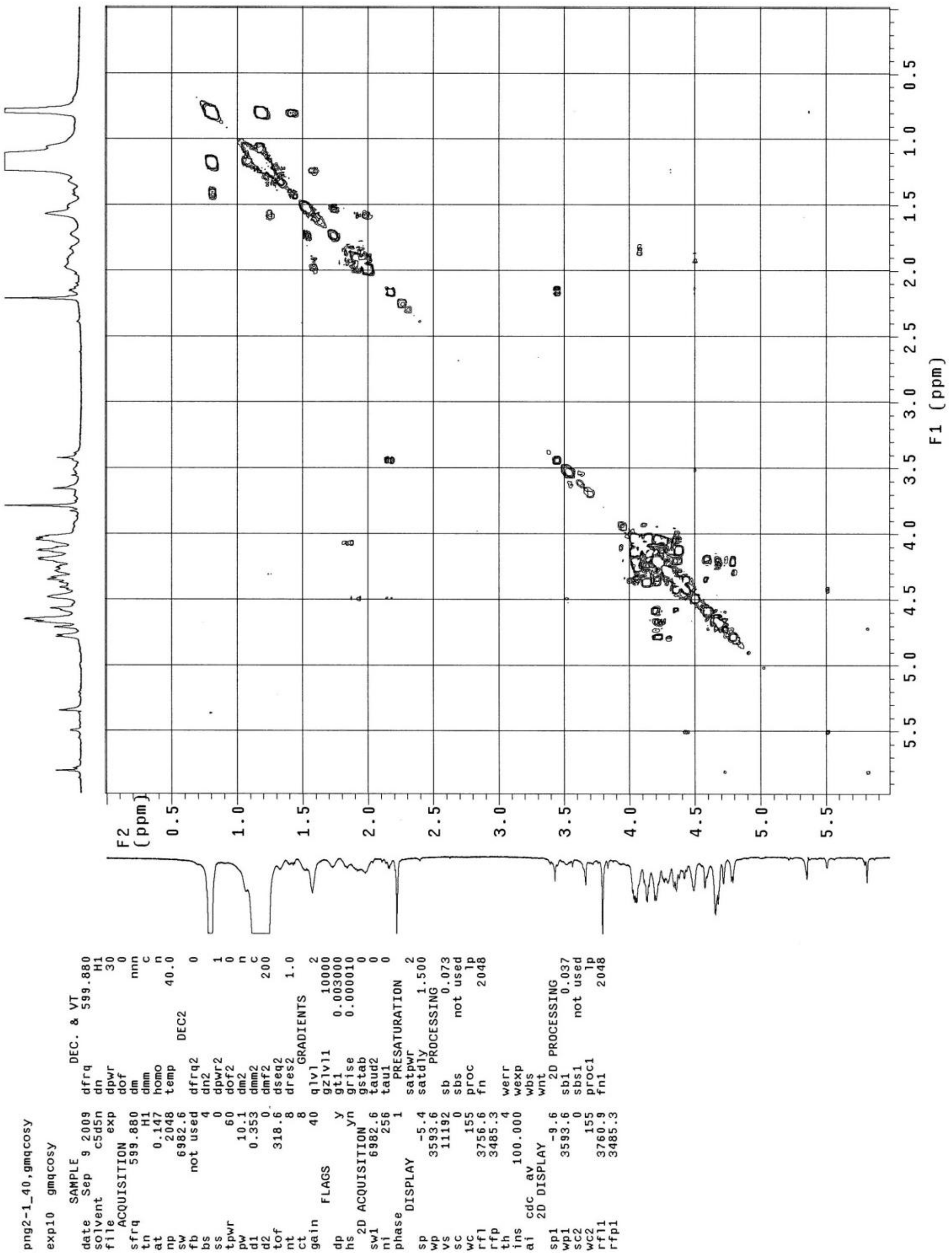

Figure S9. NOESY of PNG-1 (600 MHz, C_5D_5N/D_2O, 20/1).

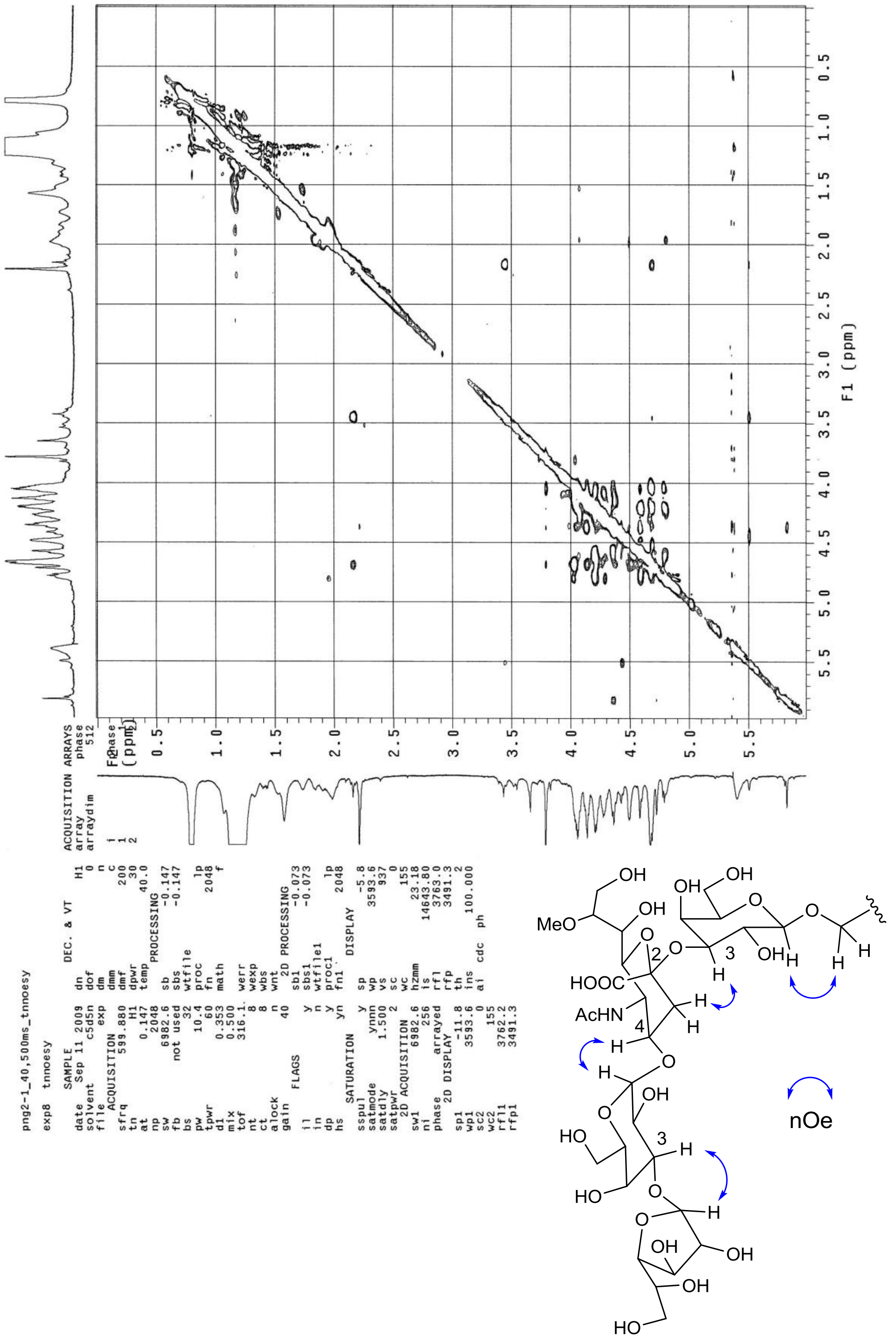

Figure S10. HSQC of PNG-2A (600 MHz, C_5D_5N/D_2O, 20/1).

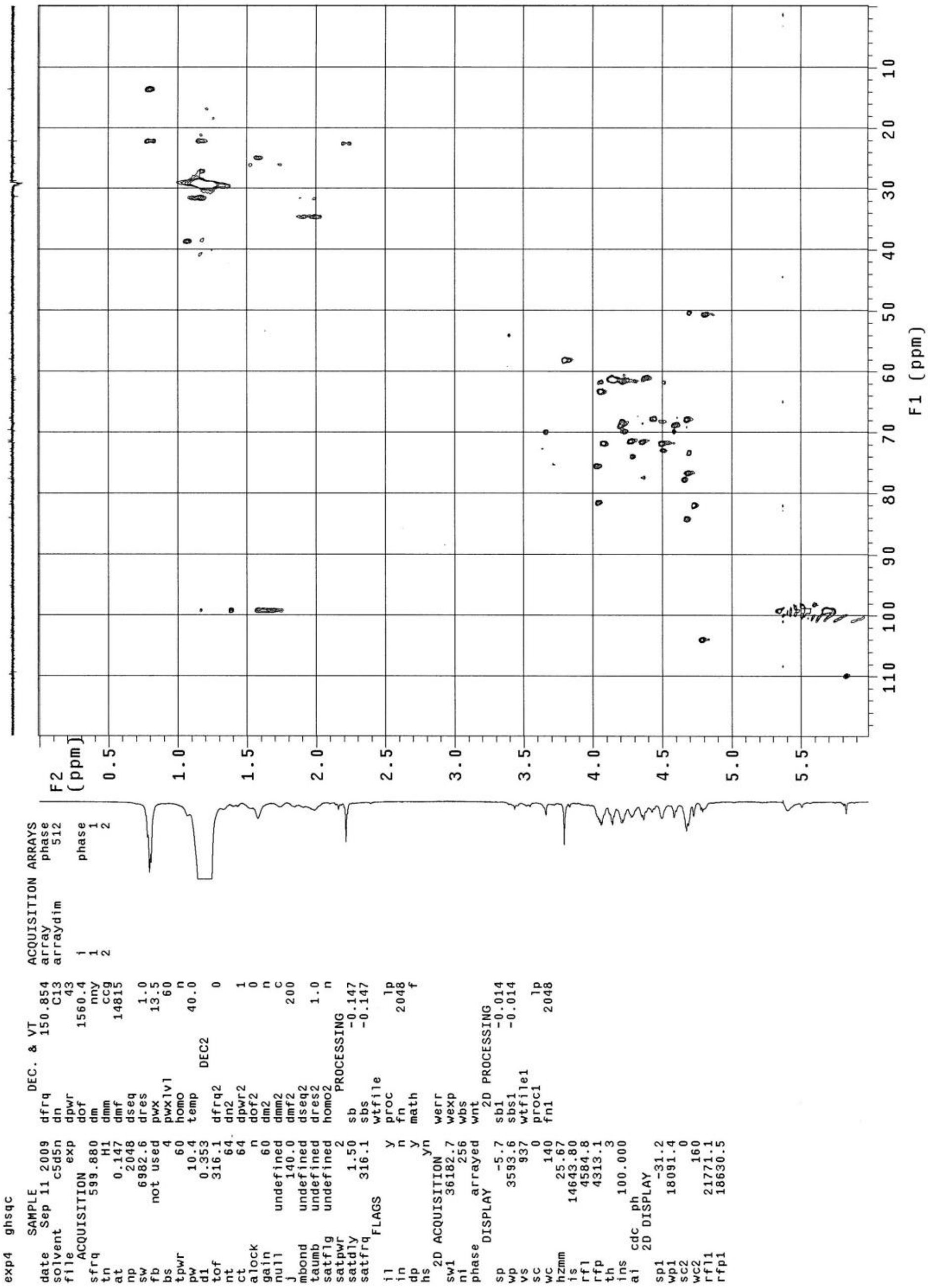

Figure S11. ^{1}H-NMR of PNG-2B (600 MHz, C_5D_5N/D_2O, 20/1).

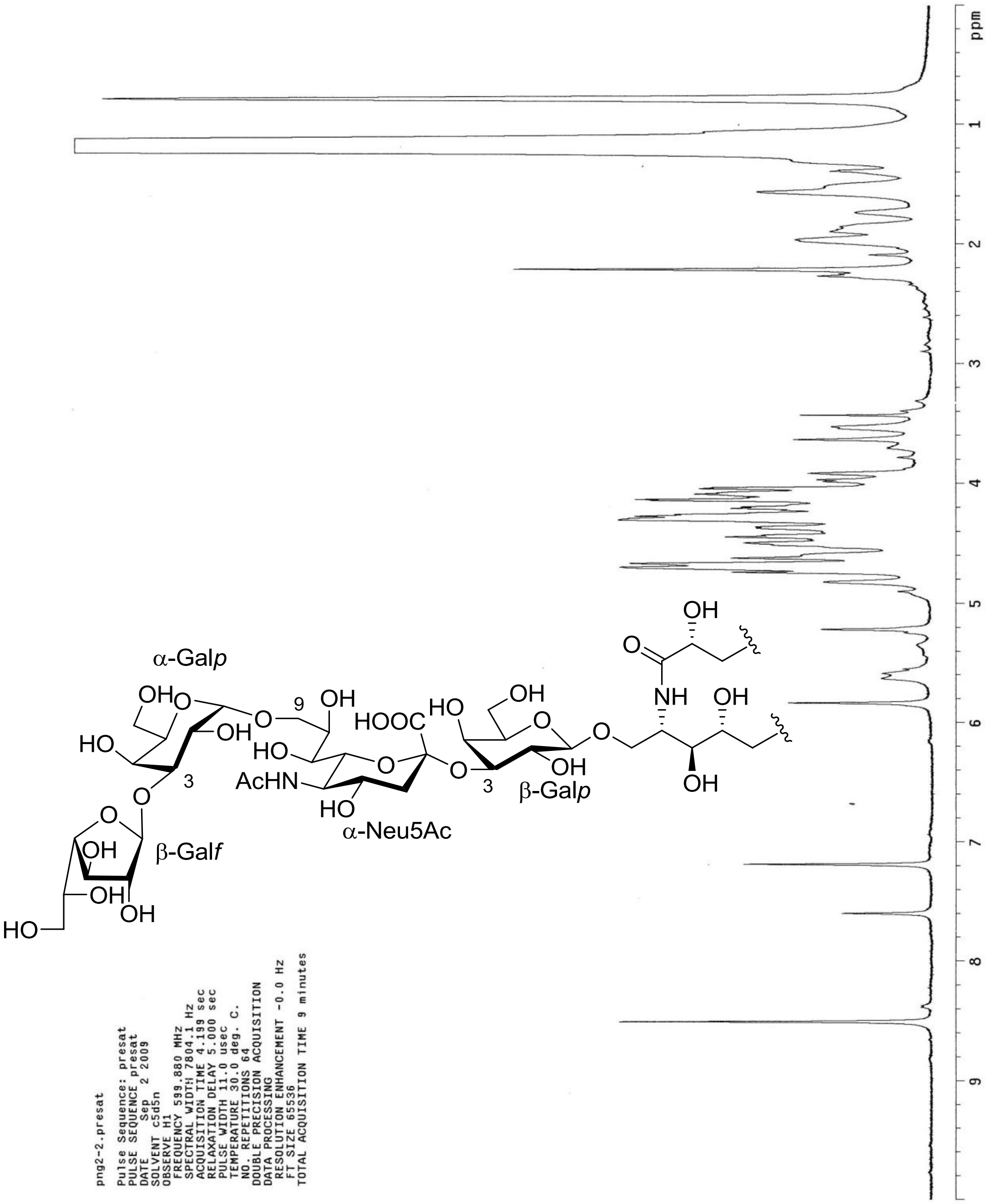

Figure S12. ^{13}C-NMR of PNG-2B (150 MHz, C_5D_5N/D_2O, 20/1).

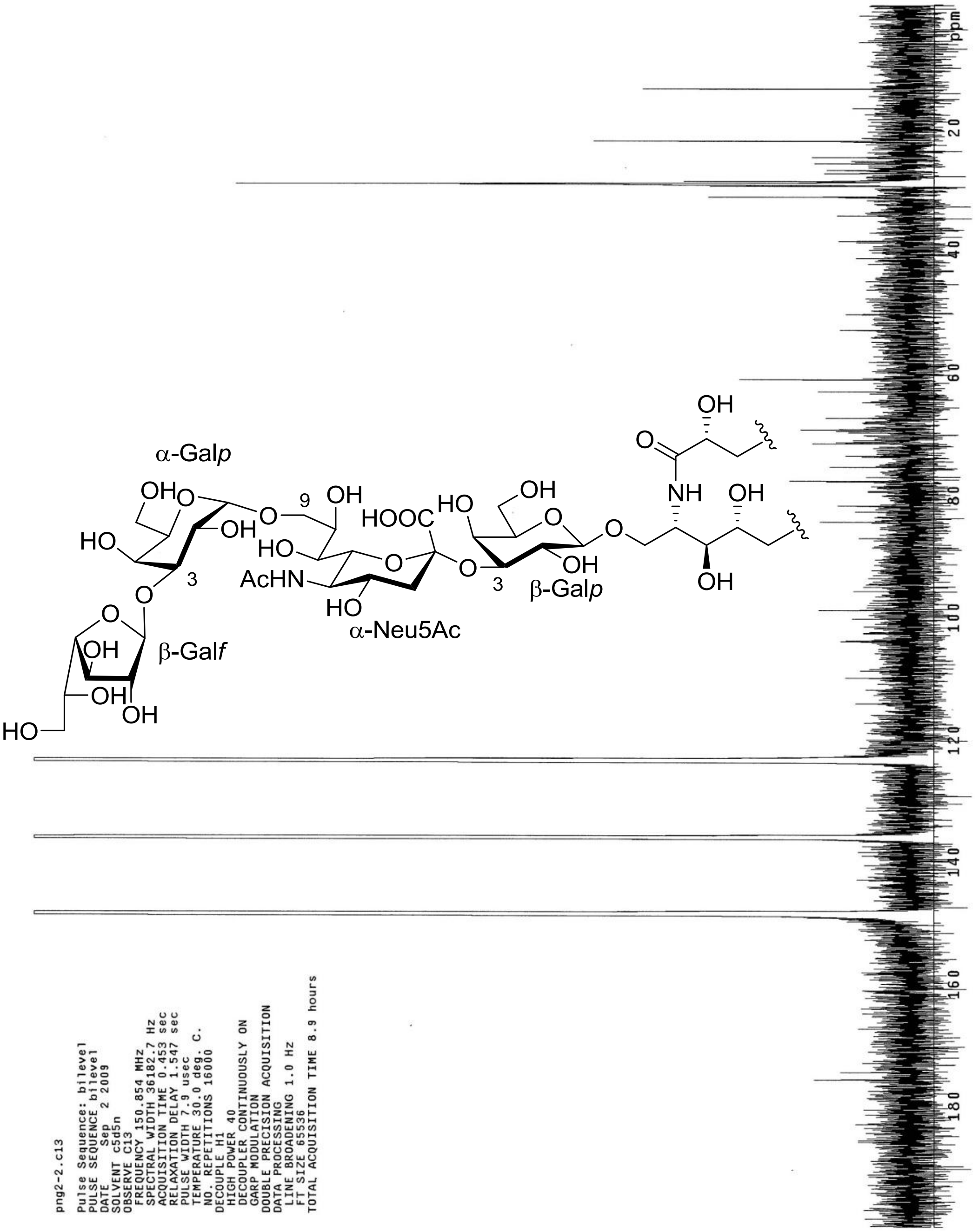

Figure S13. COSY of PNG-2B (600 MHz, C_5D_5N/D_2O, 20/1).

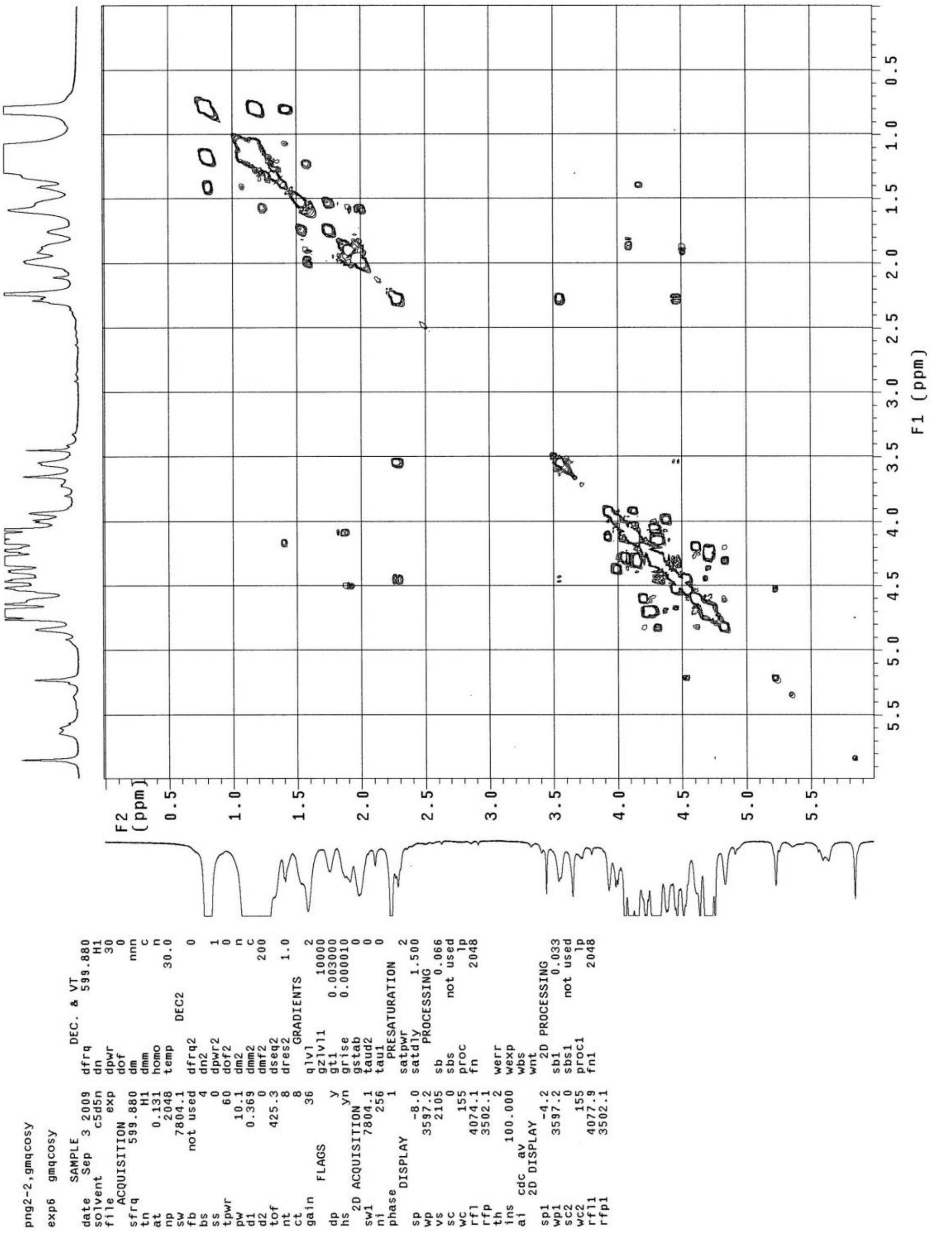

Figure S14. NOESY of PNG-2B (600 MHz, C_5D_5N/D_2O, 20/1).

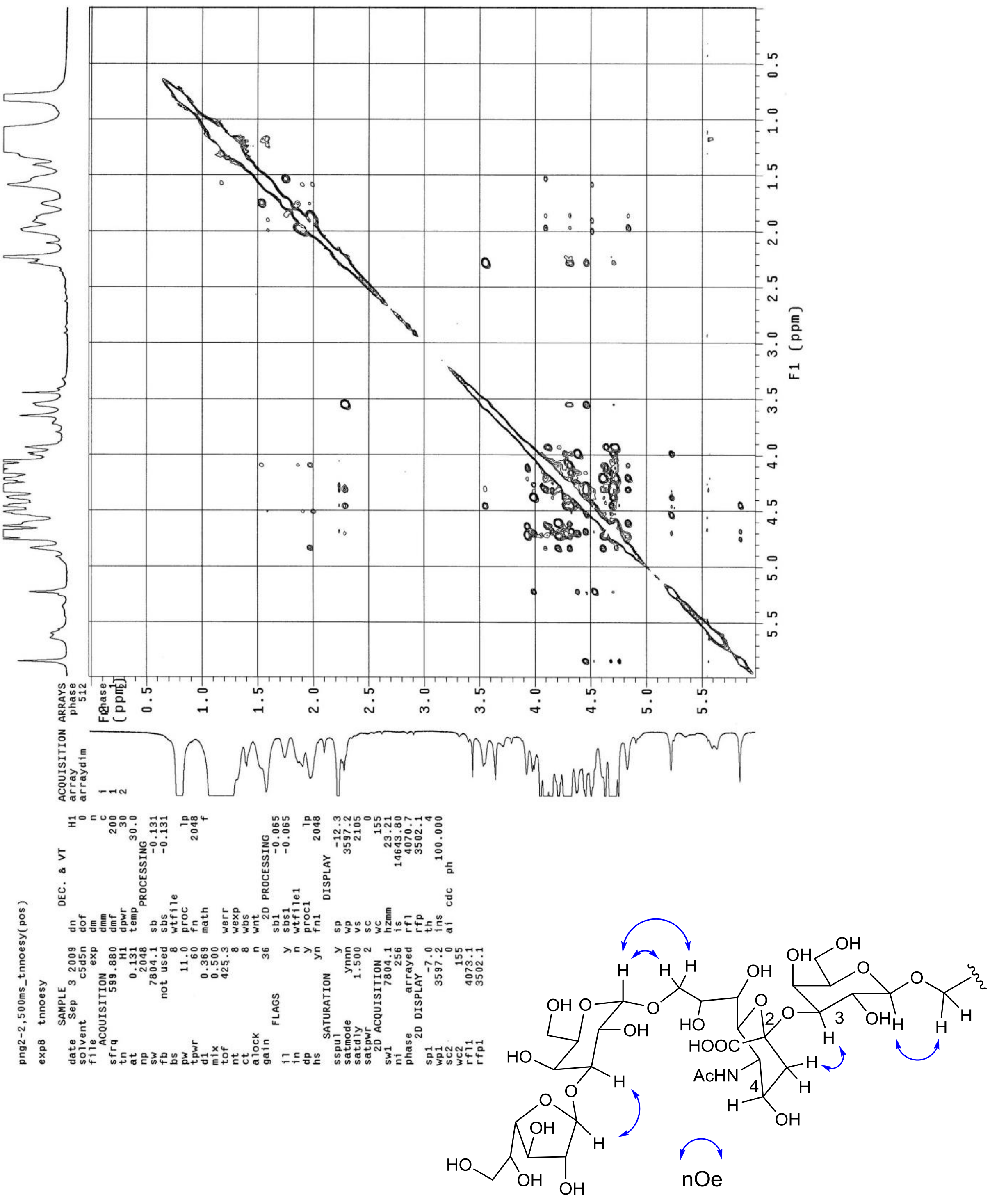

Figure S15. HSQC of PNG-2B (600 MHz, C_5D_5N/D_2O, 20/1).

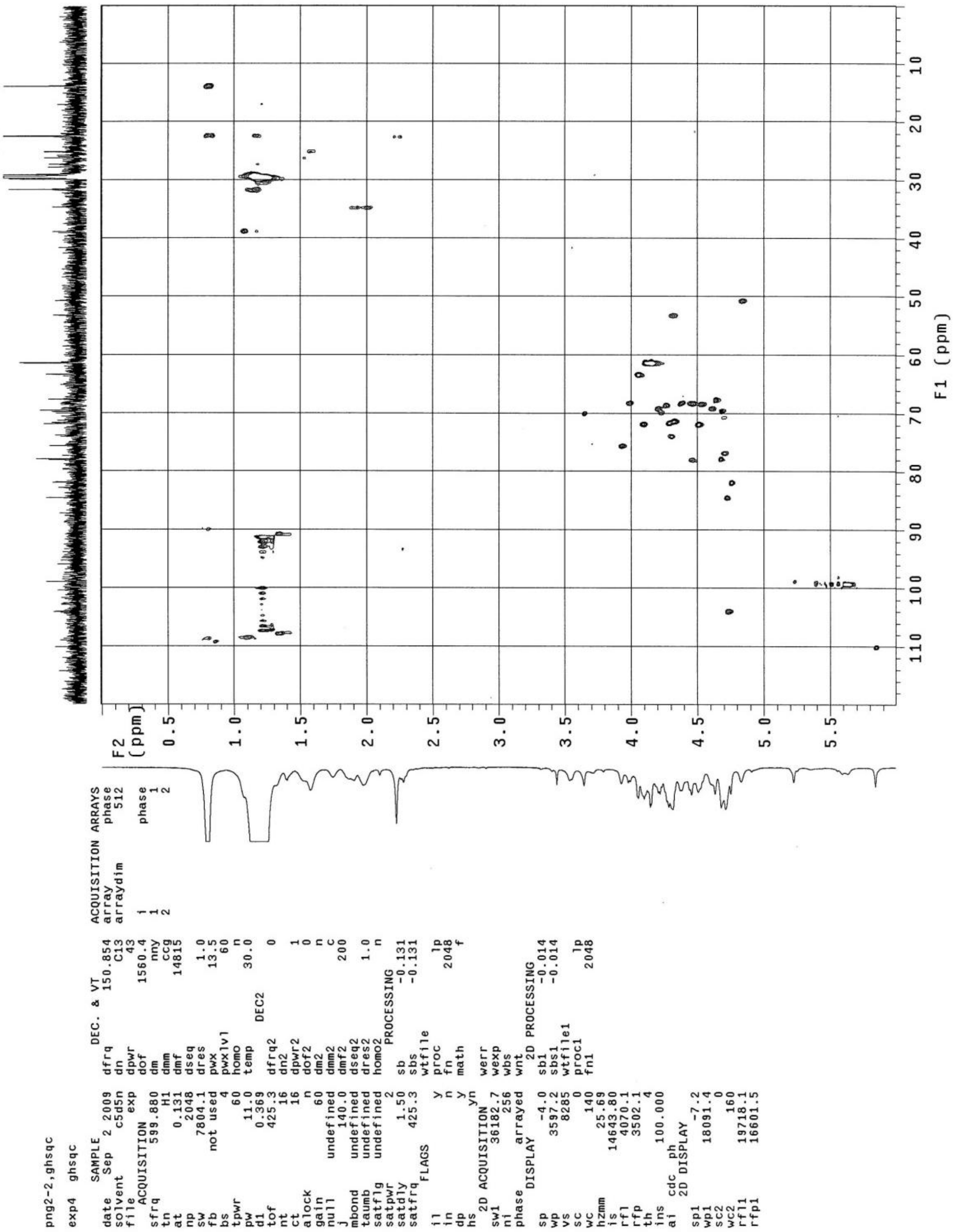

Figure S16. HMBC of PNG-2B (600 MHz, C_5D_5N/D_2O, 20/1).

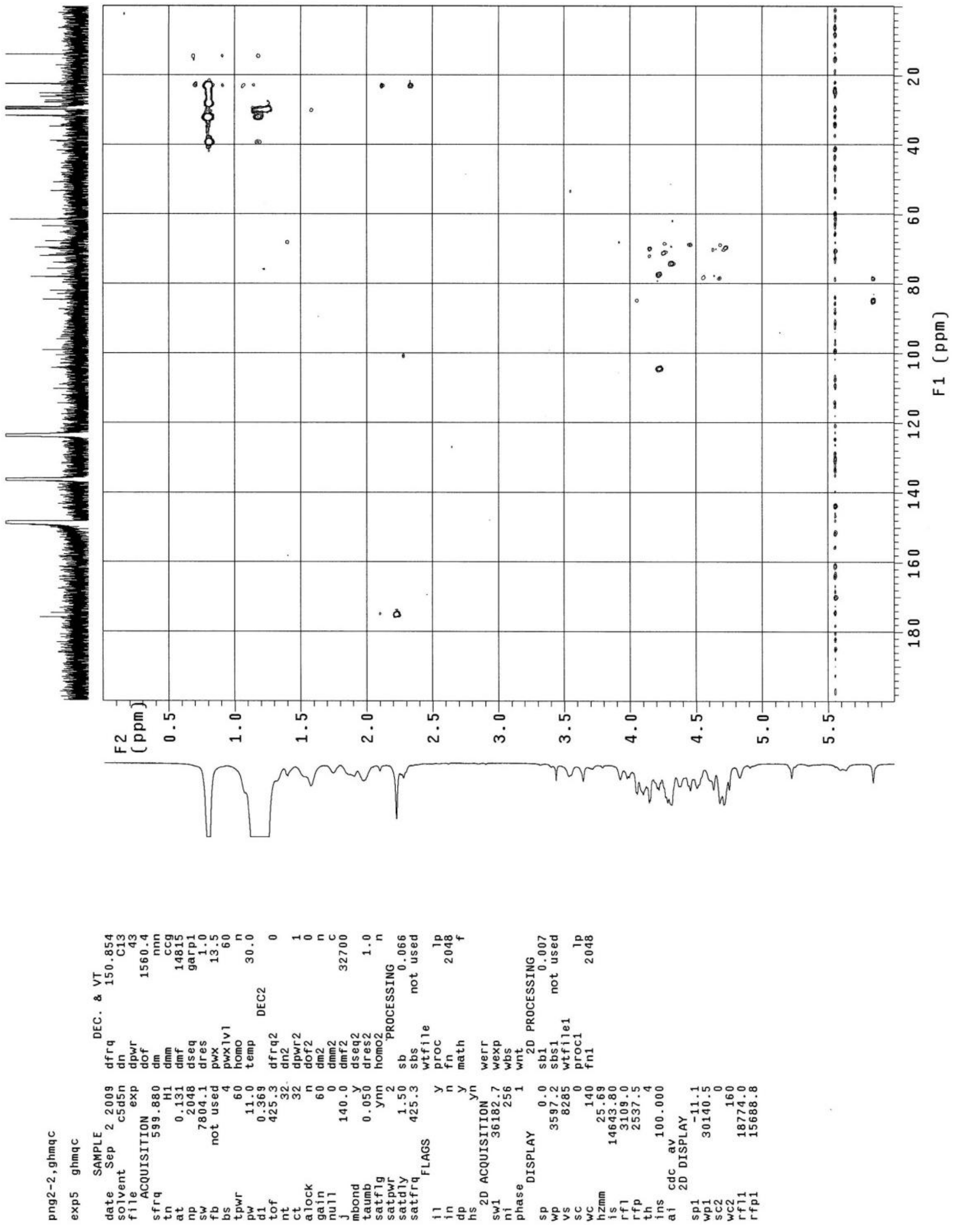

Supplement: Supplementary File 1: — PDF-Document (PDF, 2480 KB) [file marinedrugs-10-02467-s001.pdf]
